# Microbial biotechnology as a tool to restore degraded drylands

**DOI:** 10.1111/1751-7915.12832

**Published:** 2017-08-22

**Authors:** Fernando T. Maestre, Ricard Solé, Brajesh K. Singh

**Affiliations:** ^1^ Departamento de Biología y Geología Física y Química Inorgánica Escuela Superior de Ciencias Experimentales y Tecnología Universidad Rey Juan Carlos c/ Tulipán s/n 28933 Móstoles Spain; ^2^ ICREA‐Complex Systems Lab Universitat Pompeu Fabra Dr Aiguader 88 08003 Barcelona Spain; ^3^ Institut de Biologia Evolutiva CSIC‐UPF Pg Maritim de la Barceloneta 37 08003 Barcelona Spain; ^4^ Santa Fe Institute 1399 Hyde Park Road Santa Fe NM 87501 USA; ^5^ Hawkesbury Institute for the Environment Western Sydney University Penrith 2751 NSW Australia; ^6^ Global Centre for Land‐Based Innovation Western Sydney University Penrith 2751 NSW Australia

## Abstract

We briefly review how microbial biotechnology can contribute to improve activities aiming to restore degraded drylands and to combat their desertification, which are an integral part of the Sustainable Development Goal 15 of the 2030 Agenda. Microbial biotechnology offers notable promise to improve restoration actions based on the use of biocrust‐forming engineered cyanobacteria, which play key roles in maintaining ecosystem structure and functioning in drylands worldwide. Advances in our understanding of microbiome associated to biocrusts and of the signalling involved in the communication among their constituents can also potentially enhance the outcome of restoration activities in drylands.

Alterations in climate and land use, such as the intensification of grazing pressure, are main components of ongoing global environmental change that also act as major drivers of desertification, defined by the United Nations Convention to Combat Desertification as land degradation (i.e. reduction or loss of the biological or economic productivity of the land) in arid, semi‐arid and dry subhumid areas (drylands) resulting from various factors, including climatic variations and human activities. Understanding the factors driving and the consequences of land degradation and desertification, and restoring degraded dryland ecosystems constitutes key priorities for environmental agencies, land managers and stakeholders worldwide (FAO, 2015). These tasks are also extremely important to ensure global sustainability, as drylands are the largest Earth′s biome (occupy ~45% of the global terrestrial surface) and desertification is estimated to already affect over ~250 million people, mostly living in developing countries (Reynolds *et al*., 2007). This number will substantially increase in the coming decades given the forecasted increases in aridity due to climate change and to the high population growth of most developing countries.

Here, we focus on the contributions of microbial biotechnology to improve the restoration of degraded drylands and to combat desertification, which are an integral part of the Sustainable Development Goal (SDG) 15 of the 2030 Agenda, which aims to ‘protect, restore and promote sustainable use of terrestrial ecosystems, sustainably manage forests, combat desertification, and halt and reverse land degradation and halt biodiversity loss.' Actions taken to restore degraded drylands and to combat desertification strongly affect key supporting and provisioning ecosystem services for the livelihood of human populations inhabiting drylands, such as soil fertility, climate regulation and food and forage production (FAO, 2015). Therefore, they also have important implications for SDGs related to human well‐being, most notably SDG 1 (no poverty), SDG 3 (good health and well‐being), SDG 11 (sustainable cities and communities) and SDG 13 (climate action).

The provision of ecosystem services in drylands such as those named above depends fundamentally on the functioning of ecosystems, which is also strongly linked to the attributes of vascular plant and microbial communities (reviewed in Maestre *et al*., 2016). Thus, it is not surprising to find that actions to restore degraded ecosystems and to combat desertification in drylands worldwide are mainly based on the planting of vascular plants, mostly trees (FAO, 2015). These actions, however, often fail to produce the desired outcomes when they are conducted without taking into account the climatic and ecological characteristics of the area being restored, when trees are introduced using inappropriate techniques that modify its geomorphology and/or its natural vegetation, or when trees, once established, diminish water resources or reduce biodiversity (e.g. Maestre and Cortina, 2004). The failure of restoration actions can enhance erosion rates and further reduce plant cover, processes that can trigger accelerated changes leading to ecosystem collapse (Reynolds *et al*., 2007). Such *catastrophic shifts* are likely to occur in drylands, which are known to display alternative states in their structure and functioning (Maestre *et al*., 2016). Once a given external parameter, such as increasing aridity and/or grazing pressure, is slowly increased, an irreversible transition from a vegetated and functional to a degraded/desertified ecosystem can occur (Reynolds *et al*., 2007).

Ongoing increases in aridity and grazing pressure will only add further challenges to the already difficult task of restoring drylands using trees (FAO, 2015). Hence, researchers are now exploring restoration actions using communities such as biocrusts, which are formed by mosses, lichens, cyanobacteria and other microorganisms living on the soil surface of the world′s drylands (Fig. 1). Biocrusts not only are a prevalent biotic component in these ecosystems, but also strongly affect key ecosystem processes such as soil erosion, nitrogen and carbon cycling, water infiltration, run‐off generation and plant establishment and nutritional status (reviewed by Weber *et al*., 2016). Current restoration efforts using biocrusts have focused on the growth in the laboratory/greenhouse and subsequent inoculation in the field of biocrust constituents such as mosses and cyanobacteria (Weber *et al*., 2016). While there have been successful restoration experiences using these biocrust constituents in dry lands from the USA, Israel and China, the use of biocrusts in dryland restoration is far from being widespread (Weber *et al*., 2016). The difficulties to produce enough inoculum to be applied over large areas, the adaptability and growth of species grown in the laboratory to the harsh environmental conditions found in the field and the time it takes for inoculated communities to establish and effectively affect ecosystem functioning are some of the major challenges we face today to effectively restore drylands using biocrusts (Weber *et al*., 2016).

**Figure 1:**
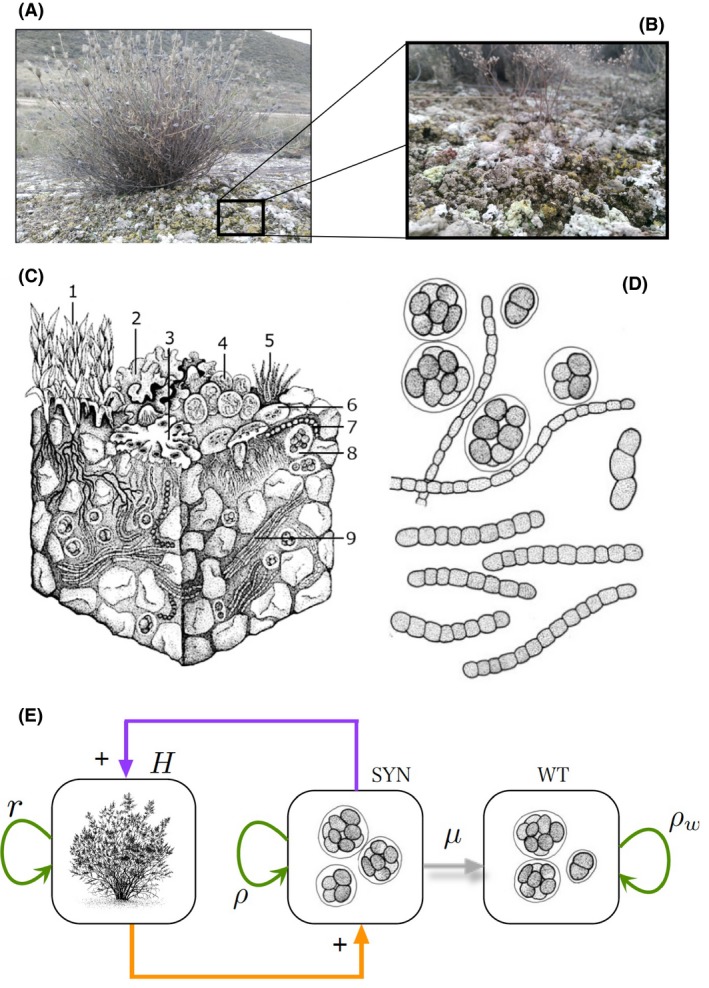
Example of a biocrust community surrounding an isolated plant in a semi‐arid ecosystem from central Spain (A). The enlarged view in B displays the detailed structure of the visible biocrust components, mostly lichens and mosses. Biocrusts are formed by a whole range of species tolerant to low moisture conditions, including mosses (1), lichens (2, 3), cyanobacteria (4, 5, 7, 9), fungi (6) and green algae (8). An example of these species is shown in D. where cells belonging to the *Nostoc* genus are represented. Biocrust‐forming cyanobacteria can be engineered to foster the restoration of degraded drylands, and thus the achievement of SDG 15, through the establishment of cooperative interactions (E). The basis of this approach is the design or modification of ecological interactions by engineering a mutualistic relation between vascular plants (H) and engineered cyanobacteria (SYN). In this scheme, SYN would have some engineered feature (such increased moisture retention by means of a synthetic exopolysaccharide) enhancing H to resist more arid conditions and creating a strong cooperative loop (blue arrows). A failure of the function to deliver would trigger a loss of the engineered constructs (grey arrow) thus recovering the original wild type (WT). Panel C redrawn from Belnap and Lange (2003).

The use of synthetic biology is being increasingly recognized as an alternative to reduce ecosystem degradation in drylands (Solé *et al*., 2015) and offers notable promise to improve restoration actions based on the use of biocrust‐forming cyanobacteria. These organisms are prevalent in dry lands worldwide, are highly resistant to desiccation and play key roles in improving soil fertility and stability, as they produce exopolysaccharides that not only increase the content of C in the soil, but also contribute to soil stabilization (Weber *et al*., 2016). Cyanobacteria have been successfully engineered in the laboratory for enhanced carbon fixation, exopolysaccharide production and biomass growth, among other properties (e.g. Kamennaya *et al*., 2015). The use of engineered biocrust‐forming cyanobacteria with these traits (vs. non‐engineered) has the potential to further increase soil fertility and to reduce soil erosion, thus accelerating the recovery of degraded drylands. These engineered cyanobacteria should be designed in a way that allows them to interact with existing organisms in predefined ways while preventing undesirable responses when released into natural conditions (Solé *et al*., 2015). This is particularly important in the case of biocrust constituents, as they are intimately linked to both vascular plants and to subsurface microbial communities, and these links should be explicitly taken into account when designing restoration schemes based on engineered cyanobacteria (Fig. 1). In this direction, theoretical studies show that some fundamental design principles could be exploited to enhance biocrust diversity and stability (Solé *et al*., 2015, 2016). An especially important one is the creation of new stable states by engineering a mutualistic loop that could be amplified by the affected partners (Fig. 1E), pushing the community far from dangerous tipping points leading to degraded ecosystem states.

Other advances in microbial biotechnology could also be used to enhance the outcome of dryland restoration activities, and thus the achievement of SDG 15. For instance, it would be possible to engineer *Pseudomonas*, a common soil bacterium found in drylands that is also a model system for many microbial engineering applications, to deliver activities of interest that could ameliorate the harsh environmental conditions typical of dryland soils and to transfer the corresponding genes to other microorganisms. In addition, *in situ* manipulations of the soil microbiome have the potential to restore microbial biomass and activity, which is usually low in degraded dryland soils, using the emerging technology of microbial cocktails and microbiome engineering (Singh and Trivedi, 2017). Such approach involves the use of a microbial consortium (probiotics) in combination with stimulants (prebiotics) that can activate dormant microbiota to enhance nutrient cycling, which in turn would encourage the activities of other biocrust partners, further improving soil fertility, stability and overall health. However, we need to address significant scientific challenges to effectively use this approach, including the characterization of the core structural and functional composition of biocrust‐associated microbiomes and the identification of the molecules involved in the communication between micro‐ and macro‐organisms forming biocrusts, as their mutual cooperation is critical for their performance under the harsh conditions characterizing degraded drylands. Technology is well advanced to characterize biocrust‐associated microbiomes, but the identification of signal molecules has been challenging because these compounds are produced in very low quantities, and current instrumentation (e.g. liquid and gas mass spectroscopy) is typically not sensitive enough to detect them at such low concentrations. However, recent studies have identified signal molecules involved in the communication between vascular plants and their associated microbiomes (Leach *et al*., 2017), and a similar approach could be employed for studying molecules responsible of communications between micro‐ and macro‐organisms forming biocrusts. If we are able to identify and synthesize these signal molecules, this will significantly advance our ability to harness this approach for restoration of degraded drylands because this will not only improve colonization and survival of biocrust communities, but will also foster nutrient cycling and make conditions more suitable for the establishment and development of vascular plants.

While numerous scientific, social and political challenges still need to be overcome before approaches based on microbial biotechnology and synthetic biology can be implemented under natural conditions, they hold an enormous potential to improve the restoration of degraded dry lands. Therefore, simultaneous advances in both research efforts and regulatory policies are needed to fully harness the potential of these emerging technologies. This would increase the suite of tools currently available to restore degraded drylands and to improve the effectiveness of actions aiming to combat global desertification and, by doing so, would facilitate the achievement of SDGs that are crucial for improving the livelihoods of the more than 400 million people living in chronic poverty in drylands worldwide.

## Conflicts of Interest

None declared.
